# Characterizing Dynamic Walking Patterns and Detecting Falls with Wearable Sensors Using Gaussian Process Methods

**DOI:** 10.3390/s17051172

**Published:** 2017-05-20

**Authors:** Taehwan Kim, Jeongho Park, Seongman Heo, Keehoon Sung, Jooyoung Park

**Affiliations:** 1 Department of Control and Instrumentation Engineering, Korea University, Sejong-Ro 2511, Sejong-City 30016, Korea; kteaw0110@korea.ac.kr (T.K.); seanpark0107@korea.ac.kr (J.P.); smheo@vadas.co.kr (S.H.); 2 Research Institute of Korean Air Lines Co., Ltd., Daejeon-City 34054, Korea; skhz89@naver.com

**Keywords:** walking, fall detection, wearable sensors, Gaussian process, dynamic model, dimensionality reduction, novelty detection, latent feature space

## Abstract

By incorporating a growing number of sensors and adopting machine learning technologies, wearable devices have recently become a prominent health care application domain. Among the related research topics in this field, one of the most important issues is detecting falls while walking. Since such falls may lead to serious injuries, automatically and promptly detecting them during daily use of smartphones and/or smart watches is a particular need. In this paper, we investigate the use of Gaussian process (GP) methods for characterizing dynamic walking patterns and detecting falls while walking with built-in wearable sensors in smartphones and/or smartwatches. For the task of characterizing dynamic walking patterns in a low-dimensional latent feature space, we propose a novel approach called auto-encoded Gaussian process dynamical model, in which we combine a GP-based state space modeling method with a nonlinear dimensionality reduction method in a unique manner. The Gaussian process methods are fit for this task because one of the most import strengths of the Gaussian process methods is its capability of handling uncertainty in the model parameters. Also for detecting falls while walking, we propose to recycle the latent samples generated in training the auto-encoded Gaussian process dynamical model for GP-based novelty detection, which can lead to an efficient and seamless solution to the detection task. Experimental results show that the combined use of these GP-based methods can yield promising results for characterizing dynamic walking patterns and detecting falls while walking with the wearable sensors.

## 1. Introduction

By incorporating a growing set of sensors and adopting machine learning technologies, wearable devices have recently become a prominent health care application domain for a variety of multi-disciplinary research areas. As a useful health care application domain along these lines, remote health monitoring systems (RHMS) (see e.g., [1,2]) have been developed to perform health care tasks such as continuous recording of health data and identifying health status. As the number of smartphones equipped with various multipurpose sensors including acceleration sensors and gyro sensors have rapidly increased, continuously recording and identifying health status from daily smartphone use is now possible, and smartphones are rapidly becoming a key intelligent device that can extract useful heath information during people’s daily activities. Many smartphone users now use smart watches, with a variety of modern functions such as sensing acceleration and rotation rate along with wireless data transmission capability. Since wearable sensors are becoming increasingly prevalent with smartphones and/or smart watches, it is possible to detect various health-related human activities in mobile device environments. Human behavior patterns that can be recognized by such means include walking and falling down. Among the relevant health care research topics, one of the most important issues is detecting falls while walking. Since falls may lead to serious injuries, automatically and promptly detecting them is particularly important.

In this paper, we are concerned with the problem of characterizing dynamic walking patterns and detecting falls while walking with wearable sensors by means of machine learning methods. Since many health issues are related to walking and/or falling, many research efforts are currently focused on the problems and questions of human walking patterns and falling. When dealing with these problems, decision making by raw sensor output data only is generally insufficient. For example, simply utilizing thresholds for sensory values from accelerators and gyros to detect falls would easily suffer from detection errors and false alarms. Recently, various machine learning methods such as artificial neural networks (ANNs), support vector machines (SVMs), and *k* nearest neighbors (kNNs) have been successfully applied to distinguishing activities such as walking and falling (see e.g., [3,4,5,6]) based on data from wearable sensors. For more related works on the subject, the reader is referred to papers such as [7,8,9,10,11,12,13]. Jeon et al. [7] proposed a method for detecting falls using two accelerometer sensor units. In their proposed method, the falls were detected based on the z-axial acceleration differences between two sensors which are located at subject’s chest and abdomen. Ellis et al. [8] addressed the problem of modeling pedestrian trajectory patterns, and proposed the use of Gaussian process regression that can model trajectory data by regressing relative motion against current position. As an application domain, the reference paper [8] considered databases collected from static surveillance cameras, and illustrated the benefit of the proposed approach for long term motion prediction. Ojetola et al. [9] proposed the use of decision trees for detecting four types of falls (forward, backward, right, and left) with wearable sensors, and showed that when applied to experimental data from eight male subjects, the proposed algorithm discriminated between activities of daily living and falls with a precision of 81% and recall of 92%. Choi et al. [10] studied on the use of machine learning algorithms for fall detection and movement classification. In their works, different machine learning algorithms were considered to identify and detect three type of normal activities (standing, walking, and lying down), four types of fall trajectories (forward, backward, left, and right), and near fall situation. Abbate et al. [11] proposed a method for the elderly people’s fall detection using smartphone and external sensor, where the falls were detected by threshold. In their method, the system produced an alarm for the acceleration higher than 3g. Also, they used the fall-like daily activities, which reduced the false alarms. Wang et al. [12] proposed an algorithm which detected five different human walking patterns from data acquired using a triaxial accelerometer. In their works, the sensor’s signal was decomposed into frequency scale by discrete Fourier transform, and then the features were classified by multi-layer perceptron neural networks. Sekine et al. [13] distinguished walking on level ground from walking on a stairway using waist acceleration signals. Their method was based on the wavelet coefficients, and the experiment was conducted with elderly people walking corridor and stairs. While machine learning methods directly handling data sets can provide reasonably good performance many cases, understanding intrinsic characteristics of human activity is often more important for attaining enhanced robustness and reliability. In this paper, we examine the problem of understanding and characterizing the intrinsic dynamics of walking patterns and detecting falls while walking with wearable sensors. For solving this problem, we utilize several Gaussian process methods for dynamic modeling, dimensionality reduction, and novelty detection. In particular, for the step of characterizing dynamic walking patterns in a latent feature space, we propose a novel approach called auto-encoded Gaussian process dynamical model (GPDM).

Gaussian processes are a branch of probabilistic kernel method closely related to non-probabilistic kernel methods such as support vector machines [14]. Recently, Gaussian processes have been successfully applied to a variety of data analysis tasks including regression, classification, and non-linear feature extraction (see e.g., [15,16,17]). One of the main advantages of the GP methods compared to non-probabilistic kernel methods is that, in their solutions, probabilistic information on uncertainty can be additionally provided. Since probabilistic uncertainty information is important in dealing with dynamic modeling, dimensionality reduction, and novelty detection problems, the GP methods are particularly useful for our purposes in this paper.

With this background in mind, we examine the problem of characterizing dynamic walking patterns and detecting falls with wearable sensors based on Gaussian process (GP) methods. In the modeling steps, we propose the combined use of the Gaussian process dynamical model (GPDM) [18,19,20] with the concept of an auto-encoder (e.g., [21,22]). The proposed combination results in the auto-encoded GPDM, a novel nonlinear dynamic structure for dimensionality reduction. Dimensionality reduction is one of the most important concepts in the field of machine learning. Well-known conventional linear dimension reduction methods include principal component analysis (PCA) and linear discriminant analysis (LDA) [23]. When the underlying structure of the problem is nonlinear and high-dimensional, nonlinear dimension reduction is more useful. Among conventional nonlinear dimension reduction methods are locally linear embedding (LLE) [24], isometric mapping (ISOMAP) [25], and kernel principal component analysis (KPCA) [26]. One of the most promising modern approaches to nonlinear dimensionality reduction is the use of auto-encoders (e.g., [21,22]), which are currently hot topics in various deep learning applications. Our auto-encoded GPDM is an extension of the GPDM [18,19,20]. In contrast to GPDM, which relies on time-consuming optimization in order to find latent features for given observation data, the GP encoder, which is derived from training the auto-encoded GPDM, can provide an intrinsic representation in low-dimensional latent space for the given observation data without resorting to optimization. For the task of detecting falls while walking, we use the GP-based novelty detection [27,28,29] for the latent samples resulting from the auto-encoded GPDM. Note that, since the latent samples generated by the training of the GPDM are recycled in our novelty detection step, the combination of these GP-based modules together are seamlessly natural.

The remainder of this paper is organized as follows. In Section 2, after providing preliminary background on Gaussian processes, we present the GP-based solutions for the problem of characterizing dynamic walking patterns and detecting falls while walking. In Section 3, the effectiveness of the GP-based solutions is illustrated by experiments, and in Section 4, the usefulness of the GP-based solutions is discussed. Finally, in Section 5, concluding remarks are provided along with topics for future studies.

## 2. Methods

### 2.1. Preliminaries

In this paper, we examine the problem of characterizing dynamic walking patterns and detecting falls while walking with built-in wearable sensors in smartphones and/or smart watches. We deal with the problem by utilizing several Gaussian process (GP) methods. Since the used GP methods are all built on Gaussian process regression (GPR) [15], we begin by introducing the concept of Gaussian process regression (GPR). For more details on Gaussian processes, readers are referred to references such as [15,16].

Gaussian processes are an example of non-parametric probabilistic models and have been successfully applied to many practical problems [15]. The Gaussian process, {f(x)}, is defined as an indexed family of random variables with index $x\in R^{d}$ such that for any finite indices, $x_{1},\cdots ,x_{N}$, the corresponding random variables, $f\left(x_{1}\right),\cdots ,f\left(x_{N}\right)$ are jointly Gaussian. In a certain stage of our application, the index space, $R^{d}$, where the index $x_{i}$ belong, will play the important role of the latent feature space. A Gaussian process {f(x)} is characterized by its mean function, m(x)=E[f(x)], and the covariance function (which is also known as the kernel function), $\kappa \left(x,x^{\prime }\right)=E\left[\left(f\left(x\right)-m\left(x\right)\right)\left(f\left(x^{\prime }\right)-m\left(x^{\prime }\right)\right)\right]$. The mean function m(x) is generally assumed to be the zero function, and the covariance function $\kappa (x,x^{\prime })$ is chosen as a class of functions parametrized by a hyper-parameter vector. The Gaussian process with the mean function m(x) and the kernel function $\kappa (x,x^{\prime })$ is denoted by $f\left(x\right)∼GP(m\left(x\right),\kappa \left(x,x^{\prime }\right))$. The concept of the Gaussian process can also be explained using weight space. In the weight space framework, f(x) is described by a linear combination of basis functions, $\sum_{j=1}^{J}w_{j}\psi_{j}\left(z\right)=\psi {\left(x\right)}^{T}w$, where ψ(x) is the feature vector, and the prior distribution of the random coefficient vector *w* is the Gaussian with a zero mean and a certain covariance matrix $\Sigma_{w}$ (i.e., $w∼N(0,\Sigma_{w})$). Note that, in the weight space framework, the mean function of f(x) satisfies $E\left[f\left(x\right)\right]=E\left[\psi {\left(x\right)}^{T}w\right]=\psi {\left(x\right)}^{T}E\left[w\right]=0$, which shows that the mean zero assumption of the Gaussian process is natural. Given the training data set $D={\left\{\left(x_{n},y_{n}\right)\right\}}_{n=1}^{N}$, we define the design matrix $Z={\left[x_{1},\cdots ,x_{N}\right]}^{T}$ as the matrix of the input points of the training data, and $T={\left[y_{1},\cdots ,y_{N}\right]}^{T}$ as the vector of the corresponding target values. Gaussian process regression often views the output *y* as generated by a zero mean Gaussian process f(x) along with an additive zero mean white Gaussian noise (i.e., y=f(x)+ϵ, where $f\left(x\right)∼GP(0,\kappa \left(x,x^{\prime }\right))$ and $\epsilon ∼N(0,\sigma_{n}^{2})$). Then, the joint distribution of the random vector $F={\left[f\left(x_{1}\right),\cdots ,f\left(x_{N}\right)\right]}^{T}$, given *Z*, can be written as

(1) p(F|Z)=N(F|0,K(Z,Z)),

where K(Z,Z) is an N×N matrix, whose (i,j)-th element is $\kappa (x_{i},x_{j})$. For notational convenience, we usually use *K* instead of K(Z,Z). Note that, by combining p(F|Z) and p(T|F) together, one can write the resultant marginal likelihood as $p\left(T|Z\right)=N\left(T|0,K+\sigma_{n}^{2}I\right)$. Hence, the log marginal likelihood for the whole training data *D* can be written as follows:

(2) $$\log p\left(T|Z\right)=-\frac{1}{2}T^{T}{\left(K+\sigma_{n}^{2}I\right)}^{-1}T-\frac{1}{2}\log\left|K+\sigma_{n}^{2}I\right|-\frac{N}{2}\log\left(2\pi \right).$$

Finding the optimal hyper-parameters can be achieved by maximizing the above log marginal likelihood function with respect to the hyper-parameters. In addition, the predictive distribution of the output $y_{*}$ for the test input point $x_{*}$ can be obtained by applying the conditional density formula for the multi-variate Gaussian distributions [15], i.e.,

(3) $$p\left(y_{*}|x_{*},D\right)=N\left(y_{*}|k_{*}^{T}{\left(K+\sigma_{n}^{2}I\right)}^{-1}T,k_{**}-k_{*}^{T}{\left(K+\sigma_{n}^{2}I\right)}^{-1}k_{*}+\sigma_{n}^{2}\right),$$

where $k_{*}={\left[\kappa \left(x_{1},x_{*}\right),\cdots ,\kappa \left(x_{n},x_{*}\right)\right]}^{T}$, and $k_{**}=\kappa \left(x_{*},x_{*}\right)$. With $\alpha ={\left[\alpha_{1},\cdots ,\alpha_{N}\right]}^{T}={\left(K+\sigma_{n}^{2}I\right)}^{-1}T$, the point estimate of the target value for the given test input point $x_{*}$ can be written as

(4) $${\hat{y}}_{*}=k_{*}^{T}{\left(K+\sigma_{n}^{2}I\right)}^{-1}T=\sum_{i=1}^{N}\alpha_{i}\kappa \left(x_{i},x_{*}\right).$$

Finally, note that Equation (4) is the canonical form of the decision function used in the kernel methods [14].

### 2.2. GP-Based Solutions for Characterizing Dynamic Walking Patterns

**Figure 1:**
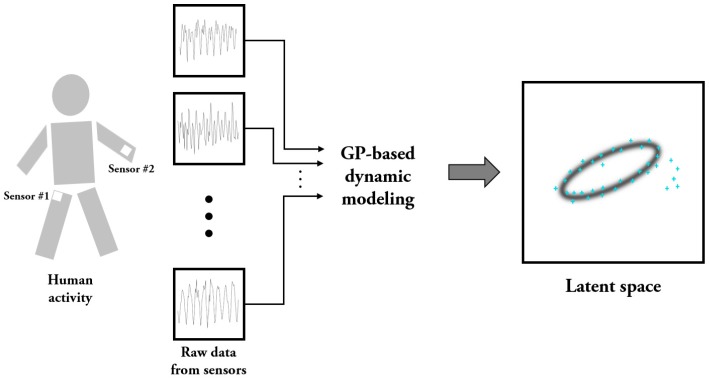
Schematic diagram for representing dynamic walking patterns in a latent space.

In this subsection, we first examine the problem of characterizing dynamic walking patterns with wearable sensors based on dynamic modeling and dimensionality reduction. There has been a wide variety of efforts for understanding dynamic walking (e.g., [30,31]). The two main approaches in these efforts are observation-based strategies and model-based strategies. In this paper, we propose a mixed strategy that utilizes the data obtained from wearable sensors together with the GP-based dynamic model for the problem. Figure 1 is the schematic diagram of our strategy for the problem, and the figure shows the configuration of the wearable sensor units in the experiments for verifying the strategy. As shown in Figure 1, we use two sets of wearable sensor units, one of which is carried in the right pocket while the remaining set is attached to the left wrist with straps. Each unit is comprised of three tri-axial devices measuring accelerations and angular velocities along three perpendicular axes. Also for robustness enhancement, we additionally consider total magnitudes [32,33] for acceleration and angular velocity. Note that these total magnitudes can provide additional measures of the degree of movement intensity. An explanation of each unit’s data set is given in Table 1. Note that the dimension of the observation space for the configuration is sixteen, i.e., $y\left(t\right)\in R^{16}$. The problem of identifying dynamic systems often relies on the auto-regressive state space model [34,35,36] described by

(5) $$y_{t+1}=h\left(y_{t},\cdots ,y_{t-L_{y}},u_{t-1},\cdots ,u_{t-L_{u}}\right)+\epsilon_{t},$$

where $y_{t}\in R^{D}$ is the output vector, $u_{t}\in R^{d}$ is the input vector, $\epsilon_{t}$ is the noise, and *h* is a nonlinear function with some universal approximation capability. However, regarding the problem of characterizing dynamic walking patterns with wearable sensors, modeling the dynamic directly with the observation vector via above auto-regressive model would be impractical because the dimension of the observed output vector, *D*, is too large. A reasonable framework for modeling the dynamics for the noise-prone high-dimensional data is to use the framework consisting of the GP state equation for low-dimensional latent space [18,19,20] and the GP output equation. This framework is often called the Gaussian process dynamical model or GPDM [18,19,20], and has the following state and output equations:

(6) $$\begin{array}{c} x_{t+1}=f_{X}\left(x_{t}\right), \end{array}$$

(7) $$\begin{array}{c} y_{t}=g_{Y}\left(x_{t}\right), \end{array}$$

where $f_{X}\left(x\right)$ and $g_{Y}\left(x\right)$ are Gaussian processes with zero means and kernel functions $\kappa_{X}\left(x,x^{\prime }\right)$ and $\kappa_{Y}\left(x,x^{\prime }\right)$, respectively (i.e., $f\left(x\right)∼GP(0,\kappa_{X}\left(x,x^{\prime }\right))$, $g\left(x\right)∼GP(0,\kappa_{Y}\left(x,x^{\prime }\right)$)). Originally, GPDM [18] was applied to the problem of 3D people tracking based on human motion capture data [18]. In this paper, we use an extended version of the GPDM for the purpose of characterizing dynamic walking patterns with wearable sensor data. For the kernel functions of the GP dynamical model, we use the following:

(8) $$\begin{array}{ccc} \kappa_{X}\left(x,x^{\prime }\right) & = & \alpha_{1}\exp(-\frac{\alpha_{2}}{2}∥x-x^{\prime }{∥}^{2})+\alpha_{3}+\alpha_{4}x^{T}x^{\prime }+\frac{\delta_{x,x^{\prime }}}{\alpha_{5}}, \end{array}$$

(9) $$\begin{array}{ccc} \kappa_{Y}\left(x,x^{\prime }\right) & = & \beta_{1}\exp(-\frac{\beta_{2}}{2}∥x-x^{\prime }{∥}^{2})+\frac{\delta_{x,x^{\prime }}}{\beta_{3}}. \end{array}$$

For the kernel functions of the decoder and transition operator, we used the similar ones with the GPDM paper [18] (i.e., we used the squared exponential (SE), constant (C), linear (LIN), and the white noise (WN) kernels for transition operator, and the squared exponential (SE) and the white noise (WN) kernels for decoder). Also for the encoder, we used the squared exponential (SE) and the white noise (WN) kernels. Note that including the linear term (LIN) is necessary when the dynamics under consideration contains a linear dynamical mode such as periodic oscillation, and the SE kernel are the most important when dealing with smooth interpolation. In addition, note that the constant term (C) is for correlating all values to some degree, and the white noise kernel (WN) is for permitting variation between variables. All of the kernel functions of Equations (8) and (9) are widely used kinds in the studies of kernel methods, and they all satisfy the Mercer law (see, e.g., [14]).

Given the matrix of observed data $Y={\left[y_{1},\cdots ,y_{N}\right]}^{T}\in R^{N\times M}$ and the matrix of the corresponding latent positions $X={\left[x_{1},\cdots ,x_{N}\right]}^{T}\in R^{N\times m}$, one can obtain the following likelihood and density by defining the kernel matrices $K_{Y}$ and $K_{X}$ with ${\left(K_{Y}\right)}_{ij}=\kappa_{Y}\left(x_{i},x_{j}\right)$ and ${\left(K_{X}\right)}_{ij}=\kappa_{X}\left(x_{i},x_{j}\right)$, respectively:

(10) $$\begin{array}{c} p\left(Y|X,\bar{\beta },W\right)=\frac{{\left|W\right|}^{N}}{\sqrt{{\left(2\pi \right)}^{ND}{\left|K_{Y}\right|}^{D}}}\exp\left(-\frac{1}{2}\mathrm{tr}\left(K_{Y}^{-1}YW^{2}Y^{T}\right)\right), \end{array}$$

(11) $$\begin{array}{c} p\left(X|\bar{\alpha }\right)=\frac{p(x_{1})}{\sqrt{{\left(2\pi \right)}^{(N-1)d}{\left|K_{X}\right|}^{d}}}\exp\left(-\frac{1}{2}\mathrm{tr}\left(K_{X}^{-1}X_{out}X_{out}^{T}\right)\right), \end{array}$$

where $X_{out}={\left[x_{2},\cdots ,x_{N}\right]}^{T}$, $X_{in}=\left[x_{1},\cdots ,x_{N-1}\right]$. Note that in Equations (10) and (11), $\bar{\alpha }$ and $\bar{\beta }$ are the sets of kernel hyper-parameters for $\kappa_{X}$ and $\kappa_{Y}$, respectively, and $W=diag(w_{1},\cdots ,w_{D})$ is the diagonal matrix of scale parameters. Note that with appropriate prior distributions over $\bar{\alpha }$, $\bar{\beta }$, and *W*, the GPDM posterior becomes

(12) $$p\left(X,\bar{\alpha },\bar{\beta },W|Y\right)\propto p\left(Y|X,\bar{\beta },W\right)p\left(X|\bar{\alpha }\right)p\left(\bar{\alpha }\right)p\left(\bar{\beta }\right)p\left(W\right).$$

**Figure 2:**
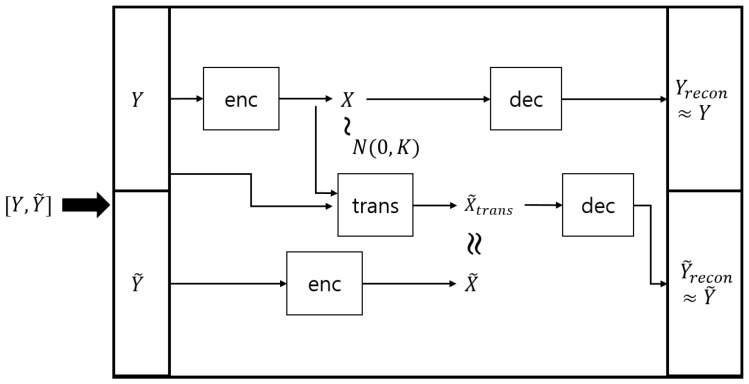
Schematic diagram of auto-encoded Gaussian process dynamical model.

**Figure 3:**
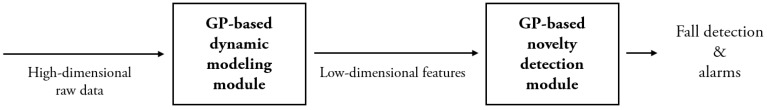
Seamless combination of GP-based walking representation and fall detection.

In the original GPDM method, the low-dimensional latent trajectories are obtained through the optimization step, which minimizes the negative log posterior of Equation (12) with respect to the latent variables. However, solving the optimization problem may be computationally expensive and limit its usefulness. In order to overcome this limitation, we propose a novel extension of the GPDM called the auto-encoded GPDM, which is essentially a combination of the GPDM with a variational auto-encoder [22]. The proposed auto-encoded GPDM consists of the GPDM decoder and the GP encoder, where the GPDM decoder is essentially the same as the original GPDM, and the aim of the newly added GP encoder is to discover the intrinsic latent representation, given the observation. More precisely, given the observation data pair $\left(Y_{1:N-1},Y_{2:N}\right)$, the GPDM decoder provides the generative model for state transition and output generation (i.e., $p_{trans}\left(X_{1:N},\alpha_{trans}\right)$ and $p_{dec}\left(Y_{1:N},\beta_{dec}|X_{1:N}\right)$), while the encoding distribution $q_{enc}\left(X_{1:N},\gamma_{enc}|Y_{1:N}\right)$ from the GP encoder approximates the true posterior distribution $p(X_{1:N}|Y_{1:N})$. For the GP encoder, we use the so-called automatic relevance determination (ARD) kernel [23] along with the inducing point method [37]. A schematic diagram for the auto-encoded GPDM is given in Figure 2. Note that the hyper-parameters of the GPDM decoder and the GP encoder are denoted by $\left\{\alpha_{trans},\beta_{dec}\right\}$ and $\gamma_{enc}$, respectively. Utilizing the strategy of variational auto-encoders (e.g., [22,38]), we can obtain the following variational objective function, $L(Y_{1:N},\alpha_{trans},\beta_{dec},\gamma_{enc})$, based on Jensen’s inequality [39] as follows:

(13) $$\begin{array}{c} \log(p\left(Y_{1:N},\alpha_{trans},\beta_{dec}\right)) \end{array}$$

(14) $$\begin{array}{cc} = & \log(\int p_{trans}\left(X_{1:N},\alpha_{trans}\right)p_{dec}\left(Y_{1:N},\beta_{dec}|X_{1:N}\right)dX_{1:N}) \end{array}$$

(15) $$\begin{array}{cc} = & \log(\int q_{enc}\left(X_{1:N},\gamma_{enc}|Y_{1:N}\right)\frac{p_{trans}\left(X_{1:N},\alpha_{trans}\right)p_{dec}\left(Y_{1:N},\beta_{dec}|X_{1:N}\right)}{q_{enc}\left(X_{1:N},\gamma_{enc}|Y_{1:N}\right)}dX_{1:N}) \end{array}$$

(16) $$\begin{array}{cc} \geq & \int q_{enc}\left(X_{1:N},\gamma_{enc}|Y_{1:N}\right)\log\left(\frac{p_{trans}\left(X_{1:N},\alpha_{trans}\right)p_{dec}\left(Y_{1:N},\beta_{dec}|X_{1:N}\right)}{q_{enc}\left(X_{1:N},\gamma_{enc}|Y_{1:N}\right)}\right)dX_{1:N} \end{array}$$

(17) $$\begin{array}{cc} = & E\left[\log p_{dec}\left(Y_{1:N},\beta_{dec}|X_{1:N}\right)\right]+\int q_{enc}\left(X_{1:N},\gamma_{enc}|Y_{1:N}\right)\log\left(\frac{p_{trans}\left(X_{1:N},\alpha_{trans}\right)}{q_{enc}\left(X_{1:N},\gamma_{enc}|Y_{1:N}\right)}\right)dX_{1:N} \end{array}$$

(18) $$\begin{array}{cc} \overset{\Delta }{=} & L(Y_{1:N},\alpha_{trans},\beta_{dec},\gamma_{enc}), \end{array}$$

where $p_{trans}$, $p_{dec}$, and $p_{enc}$ stand for transition distribution, decoder distribution, and encoder distribution, respectively; $\alpha_{trans}$, $\beta_{dec}$, and $\gamma_{enc}$ are the corresponding parameters for transition distribution, decoder distribution, and encoder distribution, respectively. Here, $X_{1:N}$ are called the latent trajectory (i.e., the state trajectory in the latent space) because they are not measured directly, but can be estimated via the proposed framework of the auto-encoded GPDM. Note that training the proposed auto-encoded GPDM for a single trajectory involves minimizing the reconstruction error for $Y_{1:N-1}$ and $Y_{2:N}$. Here, we denote $Y_{1:N-1}$ and $Y_{2:N}$ as *Y* and $\tilde{Y}$ in Figure 3 for convenience of notation. In addition, note that training based on $L(Y_{1:N},\alpha_{trans},\beta_{dec},\gamma_{enc})$ can be handled by achieving the following subgoals (see, e.g., [38]):

(19) $$\begin{array}{c} Y_{recon}\approx Y,\;{\tilde{Y}}_{recon}\approx \tilde{Y},\;{\tilde{X}}_{trans}\approx \tilde{X},\;\mathrm{and}\;q_{enc}\left(X\right)\approx N\left(0,K\right), \end{array}$$

where $Y\overset{\Delta }{=}Y_{1:N},\tilde{Y}\overset{\Delta }{=}Y_{2:N},X\overset{\Delta }{=}X_{1:N-1},\tilde{X}\overset{\Delta }{=}X_{2:N}$, and ${\tilde{X}}_{trans}$ is the output of the transition operation $trans:X_{t}\mapsto X_{t+1}$ via the state equation used in the auto-encoded GPDM framework. The first expectation on the right side of (17) is called the reconstruction loss term, which is approximated by sampling in the process of training. We call these samples in the latent space the latent samples, and utilize them efficiently in the next step for novelty detection.

**Figure 4:**
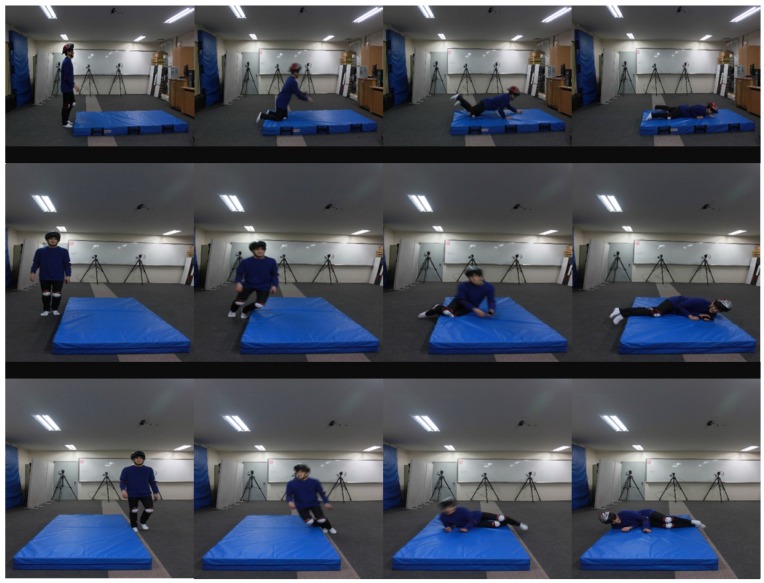
Illustrative falls.

Given any observation data, the auto-encoded GPDM is capable of providing an approximate probability density for their corresponding latent objects. Hence, in principle, one can obtain the support of the latent objects by thresholding the resultant probability density function. However, since what matters in the task of detecting abnormal objects is to gain the support of the latent objects, finding their density is stronger than necessary. In this paper, we rely on a practical alternative solution to the task. Since the training phase of the auto-encoded GPDM utilizes the latent samples generated for evaluating the reconstruction loss, we already have latent samples as a by-product of the GPDM training. Based on the latent samples for the normal class, we are able to decide whether test objects belong to normal class. This is the so-called novelty detection problem, which is often called the one-class classification problem or outlier detection problem [40,41]. Recently, GP-based novelty detection methods [27,28,29] have become available. For the task of detecting falls while walking, we utilize the predictive mean score of the GP-based novelty detection method [27] for the latent samples obtained in the training of the auto-encoded GPDM. Note that, since the latent samples generated for the training of the auto-encoded GPDM are recycled in the novelty detection step, the combination of these GP-based modules is seamlessly natural. Also, note that our method is capable of pre-impact fall detection in the sense that it can detect the falls before the body hits the ground. The schematic diagram for our combination of auto-encoded GPDM and GP based novelty detection for detecting falls is shown in Figure 3. In addition, illustrated falls are shown in Figure 4.

## 3. Experimental Results

**Figure 5:**
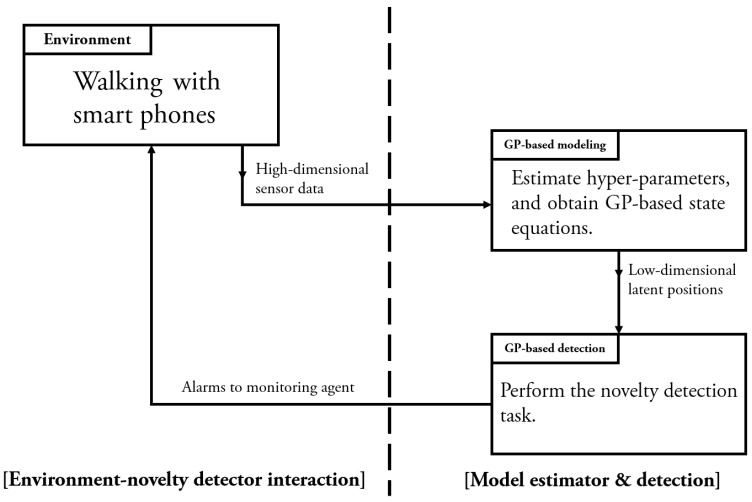
Experimental procedure for GP-based modeling and detection.

In this section, we first describe the experimental environment and data information to illustrate the proposed GP-based solutions for characterizing dynamic walking patterns and detecting falls. Following the procedure of Figure 5, we performed the experiments at the R&D Center, Korea University, with its available WiFi networks. The experiment was conducted for the five male volunteers who are 27 years old, 75 kg, 188 cm; 28 years old, 59 kg, 166 cm; 25 years old, 60 kg, 176 cm; 27 years old, 63 kg, 169 cm; 24 years old, 112 kg, 168 cm. During the entire experimental procedure, we used two smartphones: the iPhone 7 (by Apple Inc., Cupertino, CA, USA), one laptop computer: a MacBook Pro (by Apple Inc.), and one application: Matlab (mobile and PC version). The walking data were transmitted from the smartphones to the laptop computer via the WiFi networks. In our experiments, we set the sampling rate, which is the rate of transmitting data from smartphone to PC at 10 Hz. As shown in Figure 1, each test subject performed experiments with the mobile phones in the right pants pocket and the left hand. The smartphone on the left wrist was positioned facing the subject’s body, and the other one in the right pocket was positioned facing front. Based on these settings, the test subject walked the predefined courses and fell down at some random moment. Through this process, we obtained the data needed for simulating the proposed method.

In the experiment, we used the acceleration sensors and angular velocity sensors which are built into the iPhone 7. Each smartphone contains two sensors (accelerometer and gyro sensor) that can measure motion data around three orthogonal axes (*x*, *y*, *z*). We thus obtained motion data comprised of twelve features. We additionally considered the feature data with a total magnitude of 3-axes data from each sensor. As a result of acquiring the motion data and pre-processing the data, we obtained a 16-dimensional feature data (Table 1) used for our methodological model. We used the data after applying the commonly used Z-Score normalization technique.

In order to illustrate the GP-based solutions, we considered five sets of training data. In all of these training data, the observation sequence were obtained from five subjects each for 10 sec with the frequency of 10 Hz. In addition, the batch size for training was 50, and initial values of the adjustable parameters were set as: $\beta_{1:3}=\left[1,1,1\right]$, $\alpha_{1:5}=\left[1,1,1,1,1\right]$, and number of inducing points = 20.

**Figure 6:**
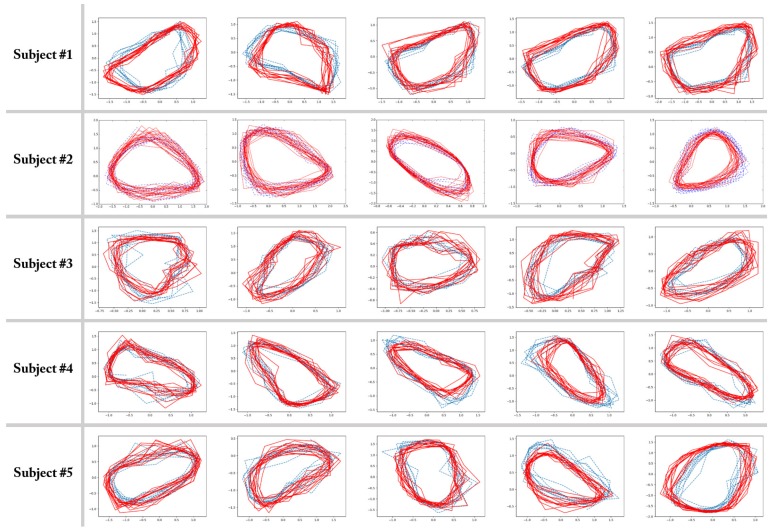
Latent trajectories learned from walking sequences using five-fold cross validation for five subjects (solid lines for test data, and dashed lines for training data). The subjects are the five male volunteers who are 27 years old, 75 kg, 188 cm; 28 years old, 59 kg, 166 cm; 25 years old, 60 kg, 176 cm; 27 years old, 63 kg, 169 cm; 24 years old, 112 kg, 168 cm. Note that there are no units for axes in latent space.

Figure 6 presents the simulation results for the five-fold cross-validation and describes the resultant trajectories in the two-dimensional latent space. The exact meaning of the pictures in the figure is as follows: in the *j*-th row, which is for the *j*-th subject, the *i*-th picture shows the latent trajectories obtained by the proposed auto-encoded GPDM procedure for the *i*-th experiment, in which the *i*-th walking data set was used as the test set, and the other four walking data sets were used as the training set for estimating the parameters of the auto-encoded GPDM. In the pictures, the solid line represents some portion of the latent trajectory provided by the GP encoder after training is completed, while the dashed lines indicate some portion of the latent trajectories of the training data sets. Figure 6 shows that the proposed auto-encoded GPDM method worked reasonably well in characterizing dynamic walking patterns in the latent space. From the cross-validation results, one can see obvious similarities between the latent trajectory of the test data and that of the training data. This indicates that the proposed auto-encoded GPDM successfully transformed a high-dimensional time series of noisy observation data signals into a time series of low-dimensional codes, and the training and test data with common characteristics indeed shared similar codes. We believe that this capability of characterizing dynamic walking patterns is of significant practical value, and can be applied to many important real world problems. In addition, we believe that the proposed methods can be deployed into current smartphone and smartwatch systems.

**Figure 7:**
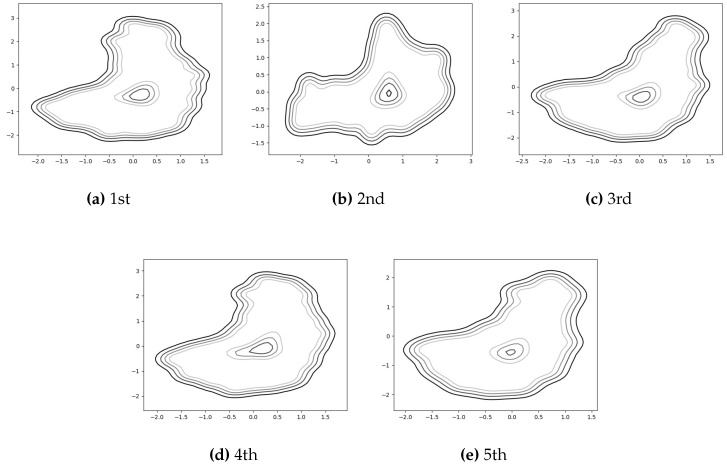
GP-based novelty detection results for a subject with the five-fold cross validation.

**Figure 8:**
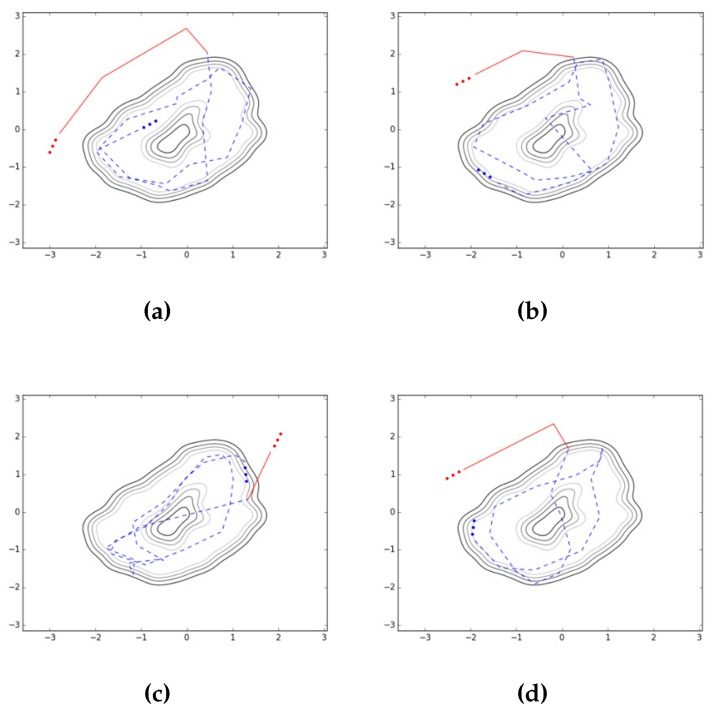
Detecting falls by GP (solid lines for trajectory after fall, and dashed lines for trajectory before fall): left, right, forward, and backward falls. Considered fall trajectories: (**a**) left, (**b**) right, (**c**) forward, (**d**) backward.

For the task of detecting falls while walking, we utilize the predictive mean score of the GP-based novelty detection method [27,28,29] for the latent samples obtained in the training of the auto-encoded GPDM. Figure 7 shows how some contours for the normal class look like for an experiment. For the contours, we utilize matplotlib.pyplot.contour [42] to show automatically-chosen-level contours for the predictive mean score. In Figure 8, some typical latent trajectories resulting from falls are shown. As indicated in the figure, one can quickly and reliably provide alarms for falls by noting the abnormal trajectory deviation from the normal region. In addition, the readers can watch the accompanying video [43], which contains an example of not only auto-encoded GPDM results for walking patterns but also detecting falls. Finally, it is one of our future works to deploy the proposed methods in current smartphone and smartwatch systems.

## 4. Discussion

In this paper, we investigated the use of Gaussian process (GP) methods for characterizing dynamic walking patterns and detecting falls while walking with built-in wearable sensors in smartphones. For the task of characterizing dynamic walking patterns in a low-dimensional latent feature space, we presented a novel approach called auto-encoded Gaussian process dynamical model, in which we combine a GP-based state space modeling method with a nonlinear dimensionality reduction method in a unique manner. Our approach is inspired by the GPDM model for human motion capture data [18]. The GPDM model is a GP-based non-parametric model for high-dimensional dynamical systems, and one of the most important strengths of the GPDM model is its capability of handling uncertainty in the model parameters [18]. Compared to the original GPDM works in [18], our works have the following two differences:

- In [18], the GPDM model was applied to the problem of 3D people tracking based on human motion capture data, whereas in this paper, we used an extended version of the GPDM for the purpose of characterizing dynamic walking patterns with wearable sensor data.
- In the original GPDM method, the low-dimensional latent trajectories were obtained through optimization, which minimizes an objective function related with the negative log posterior. The proposed auto-encoded GPDM is equipped with the GP encoder, which is capable of yielding latent representations for given observations. This capability played important roles in our finding latent trajectories for test data (e.g., solid lines in Figure 6).

**Figure 9:**
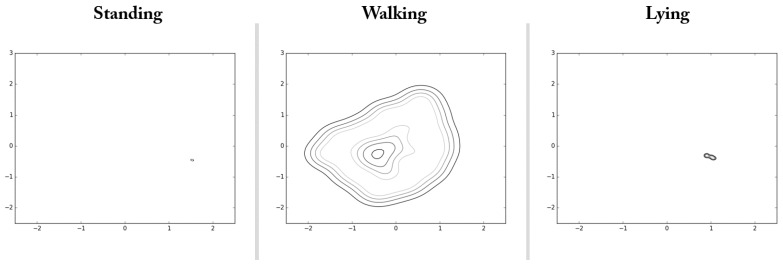
Results for intrinsic characteristics of walking, standing, and lying.

The capability of the auto-encoded GPDM can be utilized in a variety of ways. As an example along the line, we performed an experiment for the purpose of illustrating more explicitly about intrinsic characteristic of walking. For this illustration, we collected data of standing and lying down together with walking, and obtained their latent trajectories. Here for standing and lying down, we considered the postures of standing upright at a fixed position and lying down flat on the back, respectively. When collecting data for these postures, slow movements such as leaning a little forward or backward, and turning slowly a little to left or right were also allowed to imitate normal daily activities. Table 2 and Figure 9 report the training procedure and contours of the illustrative experiment conducted for the first subject of our volunteers, respectively. Contours of Figure 9 show good contrast between the considered classes of daily activities, and are capable of yielding an important intrinsic characteristic of walking. Finally, note that the above procedure can be similarly applied to the task of detecting fall while walking. In order to explore this applicability, we already performed the corresponding experiment for the first subject of our volunteers, and reported the resultant latent trajectories in Figure 8 for four cases of falls with different directions (left, right, forward, and backward). Note that the solid lines of Figure 8 showed clearly that the latent trajectories associated with the falling phase deviated significantly from those of the walking phase. It is one of our future works to perform more extensive experiments and report statistical results for the purpose of verifying the applicability.

## 5. Conclusions

In this paper, we examined the problem of characterizing dynamic walking patterns and detecting falls while walking with wearable sensors based on Gaussian process methods for dynamic modeling and novelty detection. For the wearable sensors, we used two units of wearable sensors positioned on the left wrist and in the right pocket, and from each unit, acceleration, the rate of return along three perpendicular axes, and their total magnitudes were used as input signals. For the GP-based dynamic modeling, we proposed the auto-encoded Gaussian process dynamical modeling, which can map noisy high-dimensional input space to the low-dimensional latent feature space, and the resultant latent trajectories efficiently revealed intrinsic characteristics of human dynamic walking patterns. In the training phase of the auto-encoded GPDM, the objective function was defined as the variational approximation to the negative marginal log likelihood function. According to the latent trajectories found by the GP encoder, the low-dimensional latent manifolds associated with dynamic walking patterns were smooth, while the original input signals coming from wearable sensors were often abrupt and noisy. For the task of detecting falls while walking, we used the GP-based novelty detection for the latent samples resulting from the auto-encoded GPDM. Note that, since the latent samples generated by the training for the GPDM are recycled in our novelty detection step, the combination of these GP-based modules are seamlessly natural. In addition, the experiments showed that the proposed GP approach was able to distinguish falls from normal walking. Future work yet to be done includes more extensive experiments and comparative studies, which should reveal the strengths and weaknesses of the proposed approach, and further extension of the approach in several directions. Consideration of other kinds of auto-encoders for nonlinear dimensionality reduction, and the use of a larger number of wearable sensors for the problem are some of the topics to be covered along these lines.

## Figures and Tables

**Table 1:** Each unit’s data set.

Notation	Meaning
$A_{x}$	Acceleration along the *x*-direction
$A_{y}$	Acceleration along the *y*-direction
$A_{z}$	Acceleration along the *z*-direction
$A_{T}$	Total magnitude of acceleration, i.e., $\sqrt{A_{x}^{2}+A_{y}^{2}+A_{z}^{2}}$
$\omega_{x}$	Angular velocity around the *x*-direction
$\omega_{y}$	Angular velocity around the *y*-direction
$\omega_{z}$	Angular velocity around the *z*-direction
$\omega_{T}$	Total magnitude of angular velocity, i.e., $\sqrt{\omega_{x}^{2}+\omega_{y}^{2}+\omega_{z}^{2}}$

**Table 2:** Steps for conducting and analyzing the experiment.

1: Obtain five sets of training data for each class of walking, standing, and lying.
2: Obtain five sets of test data for each class of walking, standing, and lying.
3: Train the auto-encoded Gaussian process dynamical model with the training data for walking, and fix the model.
4: Based on the fixed model, compute latent trajectory samples with the training data for each class of walking, standing, and lying.
5: Plot contours of the predictive mean score (see e.g., [27]) based on the latent trajectory samples for each class of walking, standing, and lying.
6: (optional) Perform classification for the test data.
